# Who Are the Heavy Users of Acute and Long-Term Care Services in Changhua County, Taiwan?

**DOI:** 10.1093/geroni/igaa057.2478

**Published:** 2020-12-16

**Authors:** Yuchi Young, Ye-Mei Chen, Kuo-Piao Chen, Hsiu-Hsi Chen, Wei-Jung Chang, Wan-Hsiang Hsu, Yen-Po Yeh

**Affiliations:** 1 SUNY at Albany, Rensselaer, New York, United States; 2 National Taiwan University, Taipei, Taiwan (Republic of China); 3 New York State Department of Health, Albany, New York, United States; 4 Changhua County Public Health Bureau, Changhua, Taiwan (Republic of China)

## Abstract

Identification of heavy health care users among community-dwelling older adults can lead to strategic care planning and management that positively impacts health care use and costs. This study aims to identify risk factors associated with heavy users of acute care and long-term care (LTC) in Changhua County, Taiwan. Study participants (n=8,090) included residents (age 65+) of Changhua County. Data were collected from 4/1/2017-10/26/2018. Linked hospitalization and LTC use information was provided by the Changhua County Public Health Bureau. Hospitalization was grouped into 1 vs. 2+. Univariate and multivariate logistic models will be used to address the study aim. Preliminary results show the average age was 81 years ranging from 65-105. Of this population, 56% are female, and 10% live alone. The average length of hospital stay was 9.7 days. Risk factors associated with heavy health care users identified in multivariate analysis will be presented and intervention strategies will be discussed. Part of a symposium sponsored by International Comparisons of Healthy Aging Interest Group.

